# Long non-coding RNAs and their implications in cancer epigenetics

**DOI:** 10.1042/BSR20130054

**Published:** 2013-08-30

**Authors:** Felipe C. Beckedorff, Murilo Sena Amaral, Carlos Deocesano-Pereira, Sergio Verjovski-Almeida

**Affiliations:** *Departamento de Bioquímica, Instituto de Química, Universidade de São Paulo, 05508-900 São Paulo, SP, Brazil; †Instituto Nacional de Ciência e Tecnologia em Oncogenômica, São Paulo, SP, Brazil

**Keywords:** cancer epigenetics, intergenic lncRNA, intronic lncRNA, long non-coding RNAs, regulatory RNA, RNA-guided gene silencing, ANRASSF1, antisense non-coding RNA in the RASSF1A locus, ANRIL, antisense non-coding RNA in the INK4 locus, AR, androgen receptor, ARF, ADP-ribosylation factor, CHD, chromodomain helicase DNA-binding protein, CpG, cytosine phosphodiester bond guanine, CRC, colorectal cancer, CTBP1-AS, C-terminal binding protein 1 antisense, EZH2, enhancer of zeste homologue 2, H3K27me3, histone 3 lysine 27 trymethylated, HDAC, histone deacetylase, HDM, histone demethylase, HMT, histone methyltransferase, HOTAIR, homeobox antisense intergenic RNA, HOX, homeobox, INK4a, inhibitor of cyclin-dependent kinase 4a, INK4b, inhibitor of cyclin-dependent kinase 4b, LICR, Ludwig Institute for Cancer Research, lncRNA, long non-coding RNA, lincRNA, long intergenic non-coding RNA, LSD1, lysine (K)-specific demethylase 1A, MLL, mixed-lineage leukaemia, PCAT-1, prostate cancer-associated ncRNA transcript 1, PRC, polycomb repressive complex, PSF, phosphotyrosine-binding-associated splicing factor, RASSF1A, Ras association (RalGDS/AF-6) domain family member 1 isoform A, RNAPII, RNA polymerase II, SMARCA2, SWI/SNF-related, matrix-associated, actin-dependent regulator of chromatin, subfamily A, type 2 (also known as BRM), XCI, X chromosome inactivation, XIST, X-inactive specific transcript

## Abstract

LncRNAs (long non-coding RNAs) have emerged as key molecular players in the regulation of gene expression in different biological processes. Their involvement in epigenetic processes includes the recruitment of histone-modifying enzymes and DNA methyltransferases, leading to the establishment of chromatin conformation patterns that ultimately result in the fine control of genes. Some of these genes are related to tumorigenesis and it is well documented that the misregulation of epigenetic marks leads to cancer. In this review, we highlight how some of the lncRNAs implicated in cancer are involved in the epigenetic control of gene expression. While very few lncRNAs have already been identified as players in determining the cancer-survival outcome in a number of different cancer types, for most of the lncRNAs associated with epigenetic regulation only their altered pattern of expression in cancer is demonstrated. Thanks to their tissue-specificity features, lncRNAs have already been proposed as diagnostic markers in specific cancer types. We envision the discovery of a wealth of novel spliced and unspliced intronic lncRNAs involved in epigenetic networks or in highly location-specific epigenetic control, which might be predominantly altered in specific cancer subtypes. We expect that the characterization of new lncRNA (long non-coding RNA)–protein and lncRNA–DNA interactions will contribute to the discovery of potential lncRNA targets for use in therapies against cancer.

## INTRODUCTION

According to the central dogma of molecular biology, the proteins are considered the main protagonists of cellular functions and RNAs are the intermediaries between the DNA sequences and their encoded proteins [1]. Most of the previously known classical ncRNAs (non-coding RNAs) had infrastructural functions, such as ribosomal RNAs, transporter RNAs and small RNAs involved in splicing. However, over the past decade, genome-wide transcriptional studies discovered that the eukaryotic genomes are pervasively transcribed in a large number and a wide variety of lncRNAs (long non-coding RNAs; ≥200 nt) and small ncRNAs (<200 nt), beyond the classical ncRNAs [2–5].

LncRNAs can be polyadenylated or non-polyadenylated and accumulate differentially in the nucleus and in the cytoplasm of cells [5,6]. They can be transcribed by RNAPII (RNA polymerase II) and have a 5′-cap [7]. The lncRNAs can be classified as lincRNAs (long intergenic non-coding RNAs) that are transcribed adjacent to protein-coding genes), eRNAs (enhancer RNAs that are transcribed within the enhancer regions), intronic lncRNAs (transcribed within the introns of protein-coding genes) and antisense lncRNAs (transcribed from the opposite genomic strand relative to protein-coding genes) [5,8]. These transcripts can be spliced or mono-exonic unspliced and appear to comprise the largest portion of the human non-coding transcriptome [5]; the expression level of individual lncRNAs is generally much lower than the level of expression of the typical protein-coding mRNAs [5]. Interestingly, a recent report showed that intronic RNAs constitute the major component of the mammalian ncRNA transcriptome [9], supporting the notion that intronic lncRNAs are more than just disposable parts of pre-mRNAs [9,10].

Although tens of thousands of lncRNAs are encoded in the human genome, few of them have been characterized in detail. It is already known that the lncRNAs can act via diverse mechanisms [11] and can play regulatory and structural roles in important biological processes. In almost all the examples of known functional lncRNAs, these RNA molecules associate with protein complexes, and it has been postulated that the lncRNAs may have evolved to coordinate and specialize the action of proteins in the sophisticated set of eukaryotic pathways [12]. The large amount of information accumulated in the last few years has expanded the classical roles of lncRNAs in the cells, and especially prompted an increase in complexity in the way we conceptualize RNAs, once regarded primarily as mere intermediaries of protein-coding information.

Important roles of lncRNAs have been described in epigenetic processes and this broad topic has been reviewed elsewhere [13,14]. Indeed, in a recent review, Lee [15] pointed out that lncRNAs are implicated in almost every epigenetic regulation event. It is well documented that the misregulation of epigenetic marks can result in inappropriate activation or inhibition of various genes and might lead to cancer [16]. Given the outstanding involvement of lncRNAs in epigenetic mechanisms and the altered patterns of epigenetics in cancer, the present review will discuss the participation of lncRNAs in cancer epigenetics-related processes.

### LncRNAs in cancer

Considering the wide range of roles that lncRNAs play in cellular networks [11], it is not surprising that ncRNAs have been implicated in human diseases, especially in cancer [17]. The broader involvement of lncRNAs in cancer has been extensively reviewed elsewhere [18–20]. In particular, several large-scale studies have been performed to compare the expression of lncRNAs across non-tumour and tumour samples in the past 10 years. These studies have led to the identification of several lncRNAs-based expression signatures of malignancy [21–24]. Despite the fact that some of these expression changes can be related to secondary effects of the tumour progression, experimental approaches have suggested that various lncRNAs are indeed involved in cellular transformation, thus acting as potential tumour suppressors or oncogenes, and leading to tumorigenesis [19].

In contrast with protein-coding mRNAs, which are commonly used as diagnostic and prognostic markers and are frequently expressed from multiple tissue types and cancers, most lncRNAs have tissue-specific expression patterns [5]. This peculiar characteristic of lncRNAs can offer possible benefits in terms of specificity for clinical applications. An interesting example is the lncRNA *PCA3* (prostate cancer antigen 3), which is a prostate-specific gene markedly overexpressed in prostate cancer and an established prognostic marker in prostate cancer [25]. The next expected step in clinical medicine is the use of lncRNAs in therapies, and this application may be possible in the future (reviewed in [18]).

LncRNAs that have been shown to be involved in cancer can act by diverse mechanisms, including the control of the mRNA splicing, of the mRNA translation and of the availability of miRNAs (microRNAs) to repress the mRNAs. In the present review, we will focus on those lncRNAs that act in cancer through epigenetic mechanisms.

### LncRNAs involved in cancer epigenetics

**Figure 1:**
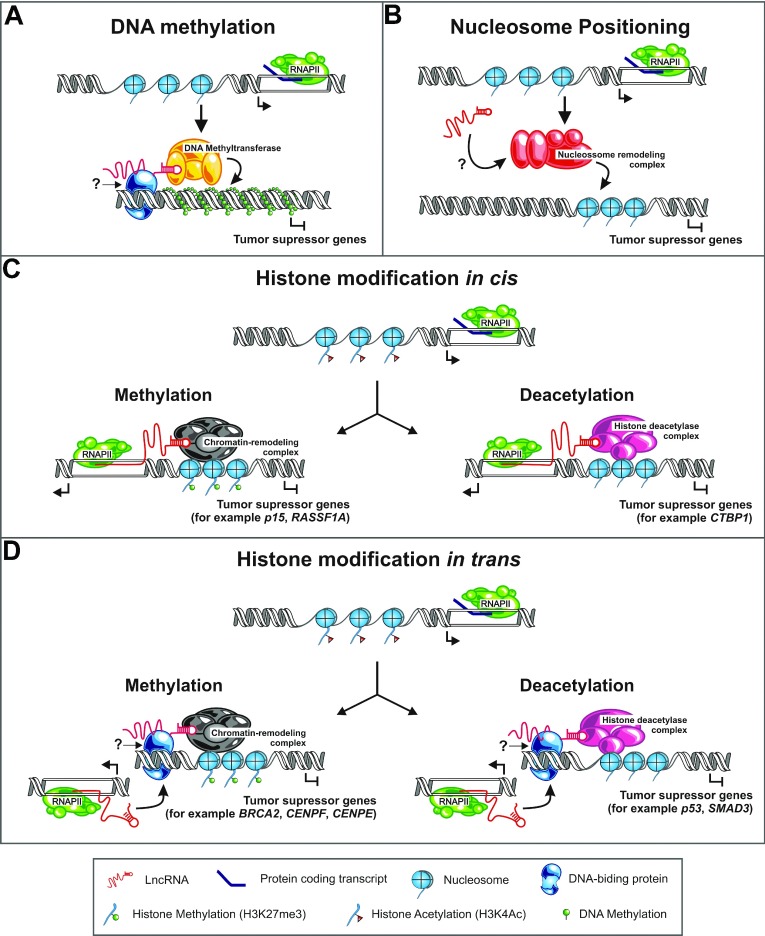
Possible and known epigenetic roles played by lncRNAs in cancer (**A**) A model of lncRNAs affecting DNA methylation. In this model, a lncRNA (red) interacts with a DNA methyltransferase and guides this protein to specific targets, leading to the methylation of the promoters and repression of tumour suppressor genes. A DNA-binding protein (dark blue) can mediate the interaction of the lncRNA with specific sites on DNA. (**B**) A model of lncRNAs changing the nucleosome positioning. A lncRNA can interact with a nucleosome remodelling complex, leading to the restructuring or dislocation of the nucleosome in specific genomic regions. An increase in the packing of the nucleosome in a region containing a tumour suppressor gene can lead to its repression. (**C**) A model of lncRNAs having in-*cis* function. In this model, an RNAPII transcribes an lncRNA (red) that can remain tethered to its transcriptional site and recruit a histone modifying enzyme (HME). This HME can lead to the methylation (left; small green circles) or to the deacetylation (right) of histones and to the subsequent silencing of tumour suppressor genes. (**D**) A model of lncRNAs acting on *trans*-regulation. In this model, an lncRNA (red) transcribed from a locus recruits a HME to a different, distant locus. This HME can lead to the methylation (left; small green circles) or to the deacetylation (right) of histones and to the subsequent silencing of tumour suppressor genes. Another possibility, not shown in the schemes, is that the lncRNAs recruit demethylases and/or acetylases to the promoter regions of oncogenes, and thus the lncRNAs might direct the transcriptional activation of such protein-coding genes.

The term epigenetics is currently used to refer to the study of heritable changes in gene expression that occur independently of modifications in the primary DNA sequence. These changes can include variable patterns of DNA methylation, nucleosome positioning and histone modifications. In the last few decades, it has been increasingly observed that the alteration of epigenetic patterns can contribute to several pathological processes, including cancer [16]. More recently, it has been recognized that the misregulation of lncRNAs is involved in cancer epigenetics [26]. Figure 1 summarizes the known and possible epigenetic mechanisms through which the lncRNAs involved in cancer can act on tumour suppressor genes, according to their roles in different categories of epigenetic modification. Another possibility, not shown in the schemes, is that the lncRNAs recruit demethylases and/or acetylases to the promoter regions of oncogenes, and thus the lncRNAs might direct the transcriptional activation of such protein-coding genes. Below, we discuss the lncRNAs that are involved in cancer and that act via epigenetic mechanisms (Table 1).

**Table 1:** Examples of functional lncRNAs in cancer epigenetics

lncRNA	Cancer type	Function/characterization	References
*ANRIL/p15AS*	Leukaemia, prostate	Binds to PRC1 and PCR2; required for the PRC2 recruitment to and silencing of *p15* tumour suppressor gene	[54,60,61]
*HOTAIR*	Breast, hepatocellular, colorectal, gastrointestinal, pancreatic	Epigenetically silences gene expression at the *HOXD* locus interacting with PCR2 and LSD1 complexes	[62,63,65]
*CTBP1-AS*	Prostate	Androgen-responsive; represses *CTBP1* expression by recruiting PSF together with histone deacetylases; promotes cell growth	[52]
*PCAT-1*	Prostate, colorectal	Inhibits BRCA2; promotes cell proliferation	[24,69]
*ANRASSF1*	Possibly prostate, breast	Binds to PRC2, represses *RASSF1A* tumour suppressor gene; increases cell proliferation	[70]
*HOTTIP*	Possibly leukaemia	Interacts with WDR5/MLL complex, which catalyses the deposition of the activating H3K4me3 mark and the transcriptional activation of the *HOXA* locus	[78]
*XIST*	Leukaemia, histiocytic sarcoma	Interacts with PRC2; epigenetically controls dosage compensation by silencing of X chromosome; suppresses cancer *in vivo*	[71]

### DNA methylation

DNA methylation has been associated with both activation and repression of the gene expression. It is known to affect mainly the cytosine residues in CpG (cytosine phosphodiester bond guanine) dinucleotides, which tend to concentrate within short CpG-rich DNA stretches called CpG islands, regions frequently located at the 5′-end of the genes that occupy approx. 60% of the human gene promoters [27]. DNA methylation can lead to gene silencing directly by preventing the recruitment of DNA-binding proteins or transcription factors to their target sites. Indirectly, it can facilitate the binding to methylated DNA of methyl-CpG-binding domain proteins, which can mediate gene repression through interaction with histone-modifying enzymes [28].

DNA methylation was one of the first epigenetic alterations identified in cancer [29]. The cancer epigenome is characterized by genome-wide hypomethylation, which can occur in several genomic sequences, such as retrotransposons, introns and repetitive elements, leading to genomic instability [30]. Additionally, hypermethylation of specific CpG islands can lead to the silencing of tumour suppressor genes involved in key cellular pathways, such as DNA repair, apoptosis and cell cycle or to the silencing of transcription factors involved in the control of these genes [31].

A lncRNA named *AS1DHRS4* (*antisense 1 dehydrogenase/reductase SDR family member 4*) is transcribed from the locus of the *DHRS4* gene and recruits DNA methyltransferases and other factors to the *DHRS4* gene cluster, inducing DNA methylation at the *DHRS4L2* promoter region [32]. Two other lncRNAs have been shown to induce either DNA methylation at specific regions of the *Kcnq1* locus [33] or demethylation at the *Sphk1* CpG island [34]; these genes are known to be related to cancer. However, a direct participation of these lncRNAs in tumorigenesis remains to be determined. Figure 1(A) shows a possible mechanism in which a lncRNA could associate with a DNA methyltransferase and guide it to the promoter region of a tumour suppressor gene, leading to the transcriptional silencing of the latter.

### Nucleosome positioning

Besides the covalent modifications printed on DNA and histones, non-covalent mechanisms play important roles in the control of gene expression by chromatin regulation. The dislocation, restructuring or destabilization of the nucleosomes are driven by ATP hydrolysis-dependent complexes that can be classified into four families: SWI/SNF, ISWI (imitation-SWI) protein, CHD (chromodomain helicase DNA-binding protein) and INO80 [35]. The positioning and remodelling of the nucleosomes are able to regulate the gene expression by altering the accessibility of regulatory DNA sequences to transcription factors and to the transcriptional machinery [36].

Unlike the roles of DNA methylation and histone modifications in the establishment and progression of cancer, the participation of nucleosome positioning in tumours is less well understood. It has been shown that mutations at and/or silencing of chromatin remodeler subunits, such as the BRG1 [also known as SMARCA4 (SWI/SNF-related, matrix-associated, actin-dependent regulator of chromatin, subfamily A, type 4)], the BRM [also known as SMARCA2 (SWI/SNF-related, matrix-associated, actin-dependent regulator of chromatin, subfamily A, type 2)] and the and SNF5 [also known as SMARCB1 (SWI/SNF-related, matrix-associated, actin-dependent regulator of chromatin, subfamily B, type 1)] subunits of the SWI/SNF complex [37] and the CHD complex [38], are present in diverse types of cancer. In plants, guiding of the ATP-dependent chromatin remodelling SWI/SNF complex by lncRNAs has been shown [39]; it is tempting to speculate that an analogous function of lncRNAs may be present in mammalians. Figure 1(B) shows the possible participation of an lncRNA in repositioning of the nucleosomes by associating with chromatin remodelling factors in the vicinity of a tumour suppressor gene.

In addition, histone variants, which are expressed outside of the S phase and are incorporated into chromatin independently from DNA replication, can influence the nucleosome occupancy and gene activity [40]. Up-regulation, down-regulation or mutations of histone variants have been associated with cancer [41]. The possible interplay between histone variants and their regulation by lncRNAs remains to be explored.

### Histone modifications

Histone modifications are catalysed by a large variety of histone-modifying enzymes, which are able to read, add or remove covalent modifications to histone proteins [42]. The final effect of the histone modifications is a change in the accessibility of the chromatin or the recruitment and/or occlusion of non-histone effector proteins, which decode the message stored in the modified histones [43].

Alterations in the expression of histone-modifying enzymes have been implicated in a wide variety of cancers. HDACs (histone deacetylases) were found to be mutated [44] or overexpressed [45] in different tumour types, which can contribute to a characteristic global loss of acetylated H4 Lys^16^ in cancer cells [46]. The balance in the acetylation levels in cancer cells is also affected by the altered expression of HATs (histone acetyl transferases), which genes were found to be mutated or deleted in several cancer types [47].

Aberrant expression of HMTs (histone methyltransferases) and HDMs (histone demethylases) has also been related to cancer by promoting an imbalance in the methylation patterns. Thus, deregulation of the HMT EZH2 (enhancer of zeste homologue 2) [48], responsible for the repressive mark H3K27me3 (histone 3 lysine 27 trymethylated), and of HMT G9a [also known as EHMT2 (histone-lysine *N*-methyltransferase)] [49], responsible for the repressive mark H3K9me3 (histone 3 lysine 9 trimethylated), leads to altered distribution of the methylation with consequent silencing of tumour suppressor genes. HDMs have also been found to be up-regulated in several cancers [50], and some of them have been proposed as targets for cancer therapy [51].

Most of the well-studied lncRNAs involved in cancer act through the recruitment of histone-modifying enzymes to target gene promoters (Table 1). Histone modification occurs in *cis*, when the lncRNA recruits the histone-modifying enzymes to the genes in the vicinity of the site of lncRNA transcription (Figure 1C). Other lncRNAs act in *trans* by recruiting the histone-modifying enzymes to different loci away from the lncRNA transcription locus (Figure 1D). Examples from the literature will be discussed in further detail below.

#### CTBP1-AS (C-terminal binding protein 1 antisense)

Recently, the identification of a new antisense lncRNA named *CTBP1-AS* has added further complexity to the regulatory epigenetic network in response to androgens in prostate cancer, in which this lncRNA acts in *cis* and in *trans* [52]. By searching for pairs sense–antisense situated in androgen-regulated tag clusters that possess AR (androgen receptor)-binding sites, the authors identified *CTBP1-AS* as an androgen-responsive lncRNA, whose expression is generally up-regulated in prostate cancer [52]. *CTBP1-AS* is located antisense to the *CTBP1*, a gene encoding a co-repressor for AR that inhibits cell growth. *CTBP1-AS* was shown to repress in *cis* the sense *CTBP1* mRNA and therefore to be associated with stimulation of cell proliferation [52]. Low levels of *CTBP1* mRNA are correlated to a poor cancer-specific survival [52]. *CTBP1* mRNA repression is mediated by binding of *CTBP1-AS* to the HDAC-Sin3A together with the repressor PSF (phosphotyrosine-binding-associated splicing factor), leading to the chromatin modification by deacetylation of the *CTBP1* promoter region in *cis* [52].

Additionally, *CTBP1-AS* acts in *trans* by participating in the recruitment and influencing the DNA-binding activity of PSF to genes involved in the cell cycle [52]. This work shows the versatility of antisense lncRNAs as players in the cell response to hormones, acting both in *cis* and in *trans* to regulate the transcriptional networks triggered by androgens. Other hormone-regulated lncRNAs have been identified [8,53], and many are likely to play a role in the regulation of expression of target genes in mammalian cells.

#### ANRIL (antisense non-coding RNA in the INK4 locus)

*ANRIL* is an antisense lncRNA that was initially described as part of the *INK4b* (inhibitor of cyclin-dependent kinase 4b)*–ARF* (*ADP-ribosylation factor*)*–INK4a* (*inhibitor of cyclin-dependent kinase 4a*) locus, which is deleted in the melanoma-neural system tumour syndrome [54]. Many studies show that the *INK4b–ARF–INK4a* locus has an important role in the regulation of the cell cycle, apoptosis and cell senescence [55,56]. Additionally, a wide range of human diseases has been associated with the aberrant expression and with SNPs (single nucleotide polymorphisms) within *ANRIL*, including several types of cancer [57].

*ANRIL* is transcribed by RNAPII and processed into alternatively spliced isoforms [58], including an unspliced transcript named *p15AS* (*p15 antisense*) that silences the tumour suppressor gene *p15* [59]. However, the mechanisms underlying *ANRIL*-mediated repression of *p15* were not elucidated [59]. More recently, it has been shown that *ANRIL* represses the *INK4b/INK4a* isoforms [60], and that this repression is mediated through the direct binding of *ANRIL* to CBX7 (chromobox homologue 7) [60], a component of the PRC1 (polycomb repressive complex 1), and to SUZ12 (suppressor of zeste 12 homologue) [61], a member of the PRC2, leading to the deposition of histone repressive marks at the locus. Together, these studies show a scenario in which *ANRIL* binds to two different PRCs and epigenetically represses the expression of genes in the *INK4b–ARF–INK4a* locus, leading to oncogenesis. Indeed, *ANRIL* has been proposed to be an oncogenic lncRNA [57].

#### HOTAIR (HOX antisense intergenic RNA)

Similar to *ANRIL*, the lincRNA *HOTAIR* is another example of possible oncogenic lncRNA. While *ANRIL* [60] regulates gene expression in *cis*, *HOTAIR* is located in the *HOXC* cluster and regulates human *HOXD* gene cluster expression in *trans* by epigenetic events [62]. *HOTAIR* was first described in fibroblasts, interacting and recruiting the PRC2, which leads to transcriptional silencing in the *HOXD* cluster through trimethylation on H3K27 [62]. Subsequent studies demonstrated that *HOTAIR* interacts with another histone-modifying complex, the LSD1 [lysine (K)-specific demethylase 1]/CoREST (repressor element 1-silencing transcription factor corepressor)/REST (repressor element 1-silencing transcription factor), which demethylates the active histone mark H3K4me3 (histone 3 lysine 4 trimethylated) [63]. *HOTAIR* functions as a scaffold for PRC2 and LSD1 [63] as well as guiding these complexes to their endogenous targets that are widespread in the genome [64].

*HOTAIR* is up-regulated in breast, colorectal, hepatocellular, gastrointestinal and pancreatic carcinomas [65–67]. *HOTAIR* was shown to be associated with metastasis in breast cancer patients [65], is a negative prognostic factor and exhibits pro-oncogenic activity in pancreatic cancer [67] and predicts tumour recurrence in hepatocellular carcinoma [66]. Importantly, it has been shown that BRCA1 (breast cancer early-onset 1) protein binding to PRC2 inhibits the binding of *HOTAIR* to the EZH2 component of PRC2, and abolishes *HOTAIR*-enhanced recruitment of PRC2 to its target *HOX* (*homeobox*) *A9* gene promoter in human breast cancer cells and fibroblasts [68]. These findings establish *HOTAIR* as the most studied lncRNA in cancer.

#### PCAT-1 (prostate cancer-associated ncRNA transcript 1)

The concept of lncRNAs as disease markers has been strongly cemented by the notable discovery of an expression signature composed of 121 prostate-specific cancer-associated intergenic lncRNAs, identified by an unbiased high-throughput sequencing of polyA+ RNA (RNA-Seq) [24]. In this work, the increased levels of lincRNA *PCAT-1* were characterized as conferring poor prognosis to prostate cancer patients [24]. *PCAT-1* is transcribed from chr8q24 and promotes prostate cancer cell proliferation by regulating target genes in *trans*, including *BRCA2* (*breast cancer early-onset 2*), *CENPF* (*centromere protein F*) and *CENPE* (*centromere protein E*) [24]. Curiously, *PCAT-1* defined a subset of aggressive cancers with low expression of the epigenetic regulator *EZH2*, a component of the *PRC2*. Besides pointing to *PRC2*-regulated selected target genes, ChIP (chromatin immunoprecipitation) revealed that the *PRC2* complex directly binds to the promoter region and represses *PCAT-1*, and RIP (RNA immunoprecipitation) showed that *PCAT-1* transcript reciprocally binds *PRC2* in a feedback inhibition loop [24].

Recently, researchers explored the correlation between *PCAT-1* expression and the progression of CRC (colorectal cancer) [69]. The authors described an increased expression of *PCAT-1* in 64% of CRC tissues from 108 cases compared with matched 81 adjacent non-tumour tissues and found that the *PCAT-1* gene copy number variation explains only a few percent of the observed *PCAT-1* overexpression [69]. Overall, these data suggest that *PCAT-1* can be used as a possible biomarker for clinical applications [24,69].

#### ANRASSF1 (antisense non-coding RNA in the RASSF1A locus)

An endogenous unspliced antisense lncRNA named *ANRASSF1* has been detected as transcribed from intronic regions at the opposite strand in the tumour-suppressor *RASSF1A* [Ras association (RalGDS/AF-6) domain family member 1 isoform A] 3p21.3 gene locus in several human cell lines [70]. *ANRASSF1* expression was higher in prostate and breast tumour cell lines compared with immortalized non-tumour lines, while the opposite pattern was found for the *RASSF1A* tumour suppressor gene [70].

*ANRASSF1* is tethered to its transcriptional site forming an RNA/DNA hybrid, binds to PRC2, and recruits this complex only to the *RASSF1A* promoter, increasing H3K27me3 repressive histone mark. Interestingly, no *ANRASSF1* effect on histone marks was detected either on the promoter of the *RASSF1C* isoform or on the promoters of four other genes in the tumour suppressor gene cluster at the 3p21.3 locus [70]. Typically, lncRNAs mediate the epigenetic modulation and silencing of imprinted gene clusters [15] or the silencing of gene clusters that are overlapped by a head-to-head natural antisense transcript [32]. *ANRASSF1* exemplified a novel highly location-specific regulatory silencing mechanism involving an antisense unspliced lncRNA, in which *ANRASSF1* repressed the expression of only the *RASSF1A* isoform overlapping the antisense transcript [70].

*ANRASSF1* overexpression decreases *RASSF1A* expression levels and increases the cell proliferation rate, whereas its silencing causes opposite effects [70]. The involvement of this potentially oncogenic lncRNA with the modulation of *RASSF1A* and tumorigenesis in cancer patients has not been studied, and further work is warranted.

#### XIST (X-inactive specific transcript)

*XIST* is a spliced and polyadenylated lncRNA with a size of 17 kb. It is one of the first identified and best-studied lncRNAs. *XIST* is typically expressed in all female somatic cells and is involved in the initiation of XCI (X chromosome inactivation) in the female cells [71]. It has been shown that *XIST* is the key regulator that triggers the XCI by binding and recruiting PRC2 first in *cis* and then spreading to several binding sites across the X chromosome, leading to the deposition of histone repressive marks in one of the X chromosome copies (see details in [72]). The expression and function of XIST is controlled by other lncRNAs such as *Jpx (X-inactive specific transcript activator)*, *RepA* (repeat A, activating *XIST*) and *TSIX* (inactivating *XIST*).

The loss of XCI and the down-regulation of *XIST* expression are frequently associated with cancers [72], but this association is strictly correlative. A recent work demonstrated that the loss of *XIST* results in X chromosome reactivation and can cause haematopoietic cancers in mice [71], presenting a direct link between X chromosome and cancer. Further studies are needed to characterize if changes in *XIST* and X chromosome are causative of cancer in humans.

## CONCLUDING REMARKS AND FUTURE PERSPECTIVES

Conspicuous patterns of expression of lncRNAs have been so far identified, especially the tissue-specificity, the low copy numbers per cell and the wide diversity of transcripts per type of tissue. Given these characteristics, it becomes obvious that the development of new tools to detect lncRNAs with higher sensitivity and at lower costs will be essential to permit the identification of patterns of expression of lncRNAs in different types of cancer and in different patient cohorts. It is expected that this better identification be translated into clinical applications such as the use of lncRNAs as prognostic and/or predictors of cancer-specific survival, as already envisioned by integrative genomic analysis [73].

LncRNAs certainly possess structural versatility allowing them to bind and interact with a number of proteins, including epigenetic regulators. The field remains vastly open for the identification of additional lncRNA-protein partners implicated in cancer. In particular, there are tens of thousands of unspliced mono-exonic antisense lncRNAs expressed from intronic regions in the human genome [4,5] that remain to be explored. We believe that the recent identification of the unspliced antisense lncRNA *ANRASSF1* as an in *cis* guide of PRC2 to a highly location-specific site [70] could be the tip of the iceberg of an epigenetic modulation mechanism driven through unspliced intronic lncRNAs that might act at highly gene-specific loci in the human genome [70]. Most probably, the activity and regulation of many other epigenetic protein complexes, besides PRC1, PRC2, LSD1 and MLL (mixed-lineage leukaemia), will prove to be dependent on specific lncRNAs. We envision that this wealth of new knowledge will be used for the application of lncRNAs as targets for selective drugs that recognize the particular structural characteristics of the lncRNAs or the lncRNA-DNA hybrids and block the recruitment of the chromatin modifying and remodeler complexes at specific gene loci. Alternatively, those lncRNAs that repress specific protein-coding genes can be interesting candidates for selective targeting by strand-specific oligonucleotides, in order to therapeutically up-regulate the protein-coding gene expression [74]. This strategy has already been tested *in vivo* [74] and could be used for lncRNAs whose expression is increased in tumour compared with non-tumour tissues and that act on tumour suppressor genes or transcription factors.

Recently, increasing attention has been given to CpG island shores that are regions of lower CpG density localized at about 2 kb of CpG island. [75]. These regions are responsible for most of the tissue-specific DNA methylation and their methylation is closely associated with transcriptional inactivation [76]. Interestingly, recent findings show that most of the aberrant DNA methylation in cancer occurs in CpG island shores instead of CpG islands located in gene promoters [76], suggesting that CpG island shores can contribute to the tumorigenic process. It remains to be determined if some lncRNAs detected as altered in tumours contribute to the deregulation of the methylation patterns of CpG island shores.

The present review is published as part of a special issue in honour of Professor Ricardo R. Brentani, who was essential in supporting the execution of, and in obtaining financial resources from Fundação de Amparo à Pesquisa do Estado de São Paulo (FAPESP) and the LICR (Ludwig Institute for Cancer Research) for the high-throughput sequencing of over 1 million ESTs (expressed sequence tags) from tumour samples of patients with more than 20 different types of cancer [77]. The work was conducted in the years 1999–2001, through the FAPESP/LICR Human Cancer Genome Project at the Hospital A.C. Camargo and at 25 other laboratories in the State of São Paulo. Brentani realized the importance of the evidence of transcription outside of the protein-coding genes, which had accumulated in the project, and he rejected the proposal from some of the project participants to keep at Hospital A.C. Camargo a collection of only a sub-set of cDNA clones, from transcripts that would be pre-determined by bioinformatics analyses to represent the protein-coding genes of interest. Rather, Brentani supported our alternative proposal, and he sponsored the establishment of a duplicate copy of the entire collection of physical cDNA clones from the project, which was located at our Department of Biochemistry, Institute of Chemistry at Universidade de São Paulo. This collection allowed us to build a custom-designed microarray enriched in cDNA probes for the putative lncRNA candidates, which was used in the pioneer work that has characterized the first intronic lncRNA expression signature correlated to the degree of tumour differentiation in prostate cancer [21].

## References

[1] Crick F. H., Barnett L., Brenner S., Watts-Tobin R. J. (1961). General nature of the genetic code for proteins. Nature.

[2] Kapranov P., Cawley S. E., Drenkow J., Bekiranov S., Strausberg R. L., Fodor S. P., Gingeras T. R. (2002). Large-scale transcriptional activity in chromosomes 21 and 22. Science.

[3] Katayama S., Tomaru Y., Kasukawa T., Waki K., Nakanishi M., Nakamura M., Nishida H., Yap C. C., Suzuki M., Kawai J. (2005). Antisense transcription in the mammalian transcriptome. Science.

[4] Nakaya H. I., Amaral P. P., Louro R., Lopes A., Fachel A. A., Moreira Y. B., El-Jundi T. A., da Silva A. M., Reis E. M., Verjovski-Almeida S. (2007). Genome mapping and expression analyses of human intronic noncoding RNAs reveal tissue-specific patterns and enrichment in genes related to regulation of transcription. Genome Biol..

[5] Djebali S., Davis C. A., Merkel A., Dobin A., Lassmann T., Mortazavi A., Tanzer A., Lagarde J., Lin W., Schlesinger F. (2012). Landscape of transcription in human cells. Nature.

[6] Kapranov P., Cheng J., Dike S., Nix D. A., Duttagupta R., Willingham A. T., Stadler P. F., Hertel J., Hackermuller J., Hofacker I. L. (2007). RNA maps reveal new RNA classes and a possible function for pervasive transcription. Science.

[7] Guttman M., Amit I., Garber M., French C., Lin M. F., Feldser D., Huarte M., Zuk O., Carey B. W., Cassady J. P. (2009). Chromatin signature reveals over a thousand highly conserved large non-coding RNAs in mammals. Nature.

[8] Wang D., Garcia-Bassets I., Benner C., Li W., Su X., Zhou Y., Qiu J., Liu W., Kaikkonen M. U., Ohgi K. A. (2011). Reprogramming transcription by distinct classes of enhancers functionally defined by eRNA. Nature.

[9] St Laurent G., Shtokalo D., Tackett M. R., Yang Z., Eremina T., Wahlestedt C., Urcuqui-Inchima S., Seilheimer B., McCaffrey T. A., Kapranov P. (2012). Intronic RNAs constitute the major fraction of the non-coding RNA in mammalian cells. BMC Genomics.

[10] Louro R., Smirnova A. S., Verjovski-Almeida S. (2009). Long intronic noncoding RNA transcription: expression noise or expression choice?. Genomics.

[11] Wang X., Song X., Glass C. K., Rosenfeld M. G. (2011). The long arm of long noncoding RNAs: roles as sensors regulating gene transcriptional programs. Cold Spring Harb. Perspect. Biol..

[12] Khalil A. M., Rinn J. L. (2011). RNA–protein interactions in human health and disease. Semin. Cell Dev. Biol..

[13] Saxena A., Carninci P. (2011). Long non-coding RNA modifies chromatin: epigenetic silencing by long non-coding RNAs. BioEssays.

[14] Mercer T. R., Mattick J. S. (2013). Structure and function of long noncoding RNAs in epigenetic regulation. Nat. Struct. Mol. Biol..

[15] Lee J. T. (2012). Epigenetic regulation by long noncoding RNAs. Science.

[16] Timp W., Feinberg A. P. (2013). Cancer as a dysregulated epigenome allowing cellular growth advantage at the expense of the host. Nat. Rev. Cancer.

[17] Esteller M. (2011). Non-coding RNAs in human disease. Nat. Rev. Genet..

[18] Spizzo R., Almeida M. I., Colombatti A., Calin G. A. (2012). Long non-coding RNAs and cancer: a new frontier of translational research?. Oncogene.

[19] Gutschner T., Diederichs S. (2012). The hallmarks of cancer: a long non-coding RNA point of view. RNA Biol..

[20] Nie L., Wu H. J., Hsu J. M., Chang S. S., Labaff A. M., Li C. W., Wang Y., Hsu J. L., Hung M. C. (2012). Long non-coding RNAs: versatile master regulators of gene expression and crucial players in cancer. Am. J. Transl. Res..

[21] Reis E. M., Nakaya H. I., Louro R., Canavez F. C., Flatschart A. V., Almeida G. T., Egidio C. M., Paquola A. C., Machado A. A., Festa F. (2004). Antisense intronic non-coding RNA levels correlate to the degree of tumor differentiation in prostate cancer. Oncogene.

[22] Tahira A. C., Kubrusly M. S., Faria M. F., Dazzani B., Fonseca R. S., Maracaja-Coutinho V., Verjovski-Almeida S., Machado M. C., Reis E. M. (2011). Long noncoding intronic RNAs are differentially expressed in primary and metastatic pancreatic cancer. Mol. Cancer.

[23] Brunner A. L., Beck A. H., Edris B., Sweeney R. T., Zhu S. X., Li R., Montgomery K., Varma S., Gilks T., Guo X. (2012). Transcriptional profiling of long non-coding RNAs and novel transcribed regions across a diverse panel of archived human cancers. Genome Biol..

[24] Prensner J. R., Iyer M. K., Balbin O. A., Dhanasekaran S. M., Cao Q., Brenner J. C., Laxman B., Asangani I. A., Grasso C. S., Kominsky H. D. (2011). Transcriptome sequencing across a prostate cancer cohort identifies PCAT-1, an unannotated lincRNA implicated in disease progression. Nat. Biotechnol..

[25] Lee G. L., Dobi A., Srivastava S. (2011). Prostate cancer: diagnostic performance of the PCA3 urine test. Nat. Rev. Urol..

[26] Morris K. V. (2009). Long antisense non-coding RNAs function to direct epigenetic complexes that regulate transcription in human cells. Epigenetics.

[27] Wang Y., Leung F. C. (2004). An evaluation of new criteria for CpG islands in the human genome as gene markers. Bioinformatics.

[28] Nan X., Ng H. H., Johnson C. A., Laherty C. D., Turner B. M., Eisenman R. N., Bird A. (1998). Transcriptional repression by the methyl-CpG-binding protein MECP2 involves a histone deacetylase complex. Nature.

[29] Feinberg A. P., Vogelstein B. (1983). Hypomethylation distinguishes genes of some human cancers from their normal counterparts. Nature.

[30] Rodriguez J., Frigola J., Vendrell E., Risques R. A., Fraga M. F., Morales C., Moreno V., Esteller M., Capella G., Ribas M. (2006). Chromosomal instability correlates with genome-wide DNA demethylation in human primary colorectal cancers. Cancer Res..

[31] Akiyama Y., Watkins N., Suzuki H., Jair K. W., van Engeland M., Esteller M., Sakai H., Ren C. Y., Yuasa Y., Herman J. G. (2003). Gata-4 and Gata-5 transcription factor genes and potential downstream antitumor target genes are epigenetically silenced in colorectal and gastric cancer. Mol. Cell Biol..

[32] Li Q., Su Z., Xu X., Liu G., Song X., Wang R., Sui X., Liu T., Chang X., Huang D. (2012). AS1DHRS4, a head-to-head natural antisense transcript, silences the DHRS4 gene cluster in *cis* and *trans*. Proc. Natl. Acad. Sci. U.S.A..

[33] Mohammad F., Pandey G. K., Mondal T., Enroth S., Redrup L., Gyllensten U., Kanduri C. (2012). Long noncoding RNA-mediated maintenance of DNA methylation and transcriptional gene silencing. Development.

[34] Imamura T., Yamamoto S., Ohgane J., Hattori N., Tanaka S., Shiota K. (2004). Non-coding RNA directed DNA demethylation of SPHK1 CpG island. Biochem. Biophys. Res. Commun..

[35] Yen K., Vinayachandran V., Batta K., Koerber R. T., Pugh B. F. (2012). Genome-wide nucleosome specificity and directionality of chromatin remodelers. Cell.

[36] John S., Sabo P. J., Thurman R. E., Sung M. H., Biddie S. C., Johnson T. A., Hager G. L., Stamatoyannopoulos J. A. (2011). Chromatin accessibility pre-determines glucocorticoid receptor binding patterns. Nat. Genet.

[37] Wilson B. G., Roberts C. W. (2011). SWI/SNF nucleosome remodellers and cancer. Nat. Rev. Cancer.

[38] Mulero-Navarro S., Esteller M. (2008). Chromatin remodeling factor CHD5 is silenced by promoter CpG island hypermethylation in human cancer. Epigenetics.

[39] Zhu Y., Rowley M. J., Bohmdorfer G., Wierzbicki A. T. (2013). A SWI/SNF chromatin-remodeling complex acts in noncoding RNA-mediated transcriptional silencing. Mol. Cell.

[40] Talbert P. B., Henikoff S. (2010). Histone variants–ancient wrap artists of the epigenome. Nat. Rev. Mol. Cell Biol..

[41] Vardabasso C., Hasson D., Ratnakumar K., Chung C. Y., Duarte L. F., Bernstein E. (2013). Histone variants: emerging players in cancer biology. Cell Mol. Life Sci..

[42] Kouzarides T. (2007). Chromatin modifications and their function. Cell.

[43] Bannister A. J., Kouzarides T. (2011). Regulation of chromatin by histone modifications. Cell Res..

[44] Ropero S., Fraga M. F., Ballestar E., Hamelin R., Yamamoto H., Boix-Chornet M., Caballero R., Alaminos M., Setien F., Paz M. F. (2006). A truncating mutation of HDAC2 in human cancers confers resistance to histone deacetylase inhibition. Nat. Genet..

[45] Zhu P., Martin E., Mengwasser J., Schlag P., Janssen K. P., Gottlicher M. (2004). Induction of HDAC2 expression upon loss of APC in colorectal tumorigenesis. Cancer Cell.

[46] Fraga M. F., Ballestar E., Villar-Garea A., Boix-Chornet M., Espada J., Schotta G., Bonaldi T., Haydon C., Ropero S., Petrie K. (2005). Loss of acetylation at Lys16 and trimethylation at Lys20 of histone H4 is a common hallmark of human cancer. Nat. Genet..

[47] Moore S. D., Herrick S. R., Ince T. A., Kleinman M. S., Dal Cin P., Morton C. C., Quade B. J. (2004). Uterine leiomyomata with t(10;17) disrupt the histone acetyltransferase morf. Cancer Res..

[48] Xu K., Wu Z. J., Groner A. C., He H. H., Cai C., Lis R. T., Wu X., Stack E. C., Loda M., Liu T. (2012). EZH2 oncogenic activity in castration-resistant prostate cancer cells is Polycomb-independent. Science.

[49] Kondo Y., Shen L., Ahmed S., Boumber Y., Sekido Y., Haddad B. R., Issa J. P. (2008). Downregulation of histone H3 lysine 9 methyltransferase G9a induces centrosome disruption and chromosome instability in cancer cells. PLoS ONE.

[50] Shi X. B., Xue L., Yang J., Ma A. H., Zhao J., Xu M., Tepper C. G., Evans C. P., Kung H. J., deVere White R. W. (2007). An androgen-regulated miRNA suppresses bak1 expression and induces androgen-independent growth of prostate cancer cells. Proc. Natl. Acad. Sci. U.S.A..

[51] Lynch J. T., Harris W. J., Somervaille T. C. (2012). Lsd1 inhibition: a therapeutic strategy in cancer?. Expert Opin. Ther. Targets.

[52] Takayama K. I., Horie-Inoue K., Katayama S., Suzuki T., Tsutsumi S., Ikeda K., Urano T., Fujimura T., Takagi K., Takahashi S. (2013). Androgen-responsive long noncoding RNA CTBP1-as promotes prostate cancer. EMBO J..

[53] Louro R., Nakaya H. I., Amaral P. P., Festa F., Sogayar M. C., da Silva A. M., Verjovski-Almeida S., Reis E. M. (2007). Androgen responsive intronic non-coding RNAs. BMC Biol..

[54] Pasmant E., Laurendeau I., Heron D., Vidaud M., Vidaud D., Bieche I. (2007). Characterization of a germ-line deletion, including the entire INK4/ARF locus, in a melanoma-neural system tumor family: identification of ANRIL, an antisense noncoding RNA whose expression coclusters with ARF. Cancer Res..

[55] Kamb A., Gruis N. A., Weaver-Feldhaus J., Liu Q., Harshman K., Tavtigian S. V., Stockert E., Day R. S., Johnson B. E., Skolnick M. H. (1994). A cell cycle regulator potentially involved in genesis of many tumor types. Science.

[56] Serrano M., Hannon G. J., Beach D. (1993). A new regulatory motif in cell-cycle control causing specific inhibition of cyclin d/CDK4. Nature.

[57] Popov N., Gil J. (2010). Epigenetic regulation of the INK4b-ARF-INK4a locus: in sickness and in health. Epigenetics.

[58] Folkersen L., Kyriakou T., Goel A., Peden J., Malarstig A., Paulsson-Berne G., Hamsten A., Hugh W., Franco-Cereceda A., Gabrielsen A. (2009). Relationship between cad risk genotype in the chromosome 9p21 locus and gene expression. Identification of eight new ANRIL splice variants. PLoS ONE.

[59] Yu W., Gius D., Onyango P., Muldoon-Jacobs K., Karp J., Feinberg A. P., Cui H. (2008). Epigenetic silencing of tumour suppressor gene p15 by its antisense RNA. Nature.

[60] Yap K. L., Li S., Munoz-Cabello A. M., Raguz S., Zeng L., Mujtaba S., Gil J., Walsh M. J., Zhou M. M. (2010). Molecular interplay of the noncoding RNA ANRIL and methylated histone H3 lysine 27 by Polycomb cbx7 in transcriptional silencing of INK4a. Mol. Cell.

[61] Kotake Y., Nakagawa T., Kitagawa K., Suzuki S., Liu N., Kitagawa M., Xiong Y. (2011). Long non-coding RNA ANRIL is required for the PRC2 recruitment to and silencing of p15(INK4b) tumor suppressor gene. Oncogene.

[62] Rinn J. L., Kertesz M., Wang J. K., Squazzo S. L., Xu X., Brugmann S. A., Goodnough L. H., Helms J. A., Farnham P. J., Segal E. (2007). Functional demarcation of active and silent chromatin domains in human HOX loci by noncoding RNAs. Cell.

[63] Tsai M. C., Manor O., Wan Y., Mosammaparast N., Wang J. K., Lan F., Shi Y., Segal E., Chang H. Y. (2010). Long noncoding RNA as modular scaffold of histone modification complexes. Science.

[64] Chu C., Qu K., Zhong F. L., Artandi S. E., Chang H. Y. (2011). Genomic maps of long noncoding RNA occupancy reveal principles of RNA-chromatin interactions. Mol. Cell.

[65] Gupta R. A., Shah N., Wang K. C., Kim J., Horlings H. M., Wong D. J., Tsai M. C., Hung T., Argani P., Rinn J. L. (2010). Long non-coding RNA HOTAIR reprograms chromatin state to promote cancer metastasis. Nature.

[66] Yang Z., Zhou L., Wu L. M., Lai M. C., Xie H. Y., Zhang F., Zheng S. S. (2011). Overexpression of long non-coding RNA HOTAIR predicts tumor recurrence in hepatocellular carcinoma patients following liver transplantation. Ann. Surg. Oncol..

[67] Kim K., Jutooru I., Chadalapaka G., Johnson G., Frank J., Burghardt R., Kim S., Safe S. (2013). HOTAIR is a negative prognostic factor and exhibits pro-oncogenic activity in pancreatic cancer. Oncogene.

[68] Wang L., Zeng X., Chen S., Ding L., Zhong J., Zhao J. C., Wang L., Sarver A., Koller A., Zhi J. (2013). BRCA1 is a negative modulator of the PRC2 complex. EMBO J..

[69] Ge X., Chen Y., Liao X., Liu D., Li F., Ruan H., Jia W. (2013). Overexpression of long noncoding RNA PCAT-1 is a novel biomarker of poor prognosis in patients with colorectal cancer. Med. Oncol..

[70] Beckedorff F. C., Ayupe A. C., Crocci-Souza R., Amaral M. S., Nakaya H. I., Soltys D. T., Menck C. F. M., Reis E. M., Verjovski-Almeida S. (2013). The intronic long noncoding RNA *ANRASSF1* recruits PRC2 to the *RASSF1A* promoter, reducing the expression of *RASSF1A* and increasing cell proliferation. PLoS Genet..

[71] Yildirim E., Kirby J. E., Brown D. E., Mercier F. E., Sadreyev R. I., Scadden D. T., Lee J. T. (2013). Xist RNA is a potent suppressor of hematologic cancer in mice. Cell.

[72] Froberg J. E., Yang L., Lee J. T. (2013). Guided by RNAs: X-inactivation as a model for lncRNA function. J. Mol. Biol..

[73] Du Z., Fei T., Verhaak R. G., Su Z., Zhang Y., Brown M., Chen Y., Liu X. S. (2013). Integrative genomic analyses reveal clinically relevant long noncoding RNAs in human cancer. Nat. Struct. Mol. Biol..

[74] Wahlestedt C. (2013). Targeting long non-coding RNA to therapeutically upregulate gene expression. Nat. Rev. Drug Discov..

[75] Irizarry R. A., Ladd-Acosta C., Wen B., Wu Z., Montano C., Onyango P., Cui H., Gabo K., Rongione M., Webster M. (2009). The human colon cancer methylome shows similar hypo- and hypermethylation at conserved tissue-specific CpG island shores. Nat. Genet..

[76] Doi A., Park I. H., Wen B., Murakami P., Aryee M. J., Irizarry R., Herb B., Ladd-Acosta C., Rho J., Loewer S. (2009). Differential methylation of tissue- and cancer-specific CpG island shores distinguishes human induced pluripotent stem cells, embryonic stem cells and fibroblasts. Nat. Genet..

[77] Brentani H., Caballero O. L., Camargo A. A., da Silva A. M., da Silva W. A., Dias Neto E., Grivet M., Gruber A., Guimaraes P. E., Hide W. (2003). The generation and utilization of a cancer-oriented representation of the human transcriptome by using expressed sequence tags. Proc. Natl. Acad. Sci. U.S.A..

[78] Wang K. C., Yang Y. W., Liu B., Sanyal A., Corces-Zimmerman R., Chen Y., Lajoie B. R., Protacio A., Flynn R. A., Gupta R. A. (2011). A long noncoding RNA maintains active chromatin to coordinate homeotic gene expression. Nature.

